# Silent spinal epidural abscess due to facet joint arthritis

**DOI:** 10.1016/j.idcr.2023.e01887

**Published:** 2023-08-28

**Authors:** Yuta Nakamura, Takahiro Namba, Momoko Sakurai, Masato Yasui

**Affiliations:** Department of Pediatrics, Fukuyama City Hospital, Fukuyama, Japan

**Keywords:** Spinal epidural abscess, Facet joint arthritis, Fibrinogen, D-dimer, Magnetic resonance imaging, Pediatric

## Abstract

Spinal epidural abscess (SEA) is an abscess that forms between the dura mater and vertebrae. SEA is characterized by back pain and neuropathy associated with fever, of which early diagnosis and treatment are necessary to avoid irreversible neurological sequelae. However, its diagnosis is often difficult because specific symptoms are rarely present in the early stages of the disease. A 25-month-old boy, healthy by nature and free of risk factors, was referred and admitted for fever symptoms only, without back pain or neurological symptoms. We focused on the residual activation of the coagulation-fibrinolytic system, which was contrary to the response to therapy, and were able to establish a diagnosis of SEA. After the initiation of antibiotics, the patient responded well to treatment and made a mild recovery without the need for surgical intervention. To date, there are no reported cases of SEA with only febrile symptoms without localized spinal cord tenderness. SEA is easily overlooked and should be considered in the differential diagnosis of pediatric fever of unknown origin. Although imaging studies have drawbacks, such as radiation exposure and sedation, they should be immediately performed if SEA is suspected.

## Introduction

Spinal epidural abscess (SEA) is the collection of pus and inflammatory tissue that forms between the dura mater and vertebrae [1], [2], [3], [4]. SEA is primarily a disease in older adults with an underlying disease but may be exhibited very rarely in children [1], [4], [5], [6]. Due to its infrequency and the lack of specificity of manifestations, SEA diagnosis is often delayed [5], [6], [7]. However, prompt diagnosis and treatment are necessary to prevent devastating neurological sequelae [1], [4], [5]. In the present report, we diagnosed SEA in an affected child with no risk factors and only febrile symptoms and successfully treated him with antibiotics without neurological complications.

## Case

A 25-month-old boy was referred to our hospital following a 4-day fever. The patient had no history of infection, trauma, surgery, or tuberculosis or recent history of receiving injections. He had completed all required vaccinations to this point. On admission, his temperature, blood pressure, heart rate, and respiratory rate were 38.8 °C, 86/48 mmHg, 120 beats/min, and 20 breaths/min, respectively. The patient did not experience headache or abdominal or back pain and could sit and walk without discomfort. Neurological examination did not reveal any findings suggestive of meningitis. His chest radiograph revealed no abnormalities. Laboratory tests revealed white blood cell count, 14,200/mL (69.2% neutrophils); C-reactive protein (CRP), 10.8 mg/dL (normal range: < 0.2 mg/dL); procalcitonin, 0.70 ng/mL (< 0.05 μg/L); fibrinogen, 1175 mg/dL (200–400 mg/dL); fibrin/fibrinogen degradation products (FDP), 14.0 μg/mL (1.0–10.0 μg/mL); and D-dimer, 5.1 μg/mL (0.15–1.0 μg/mL). Although the patient had received oral antibiotics before referral, blood and urine cultures were routinely performed. The site of inflammation and causative organism were not identified. However, a severe infection was suspected. Therefore, we decided to first treat the patient with a broad-spectrum antibiotic at the recommended dose for severe infection. Meropenem (90 mg/kg/day) was intravenously administered.

**Fig. 1:**
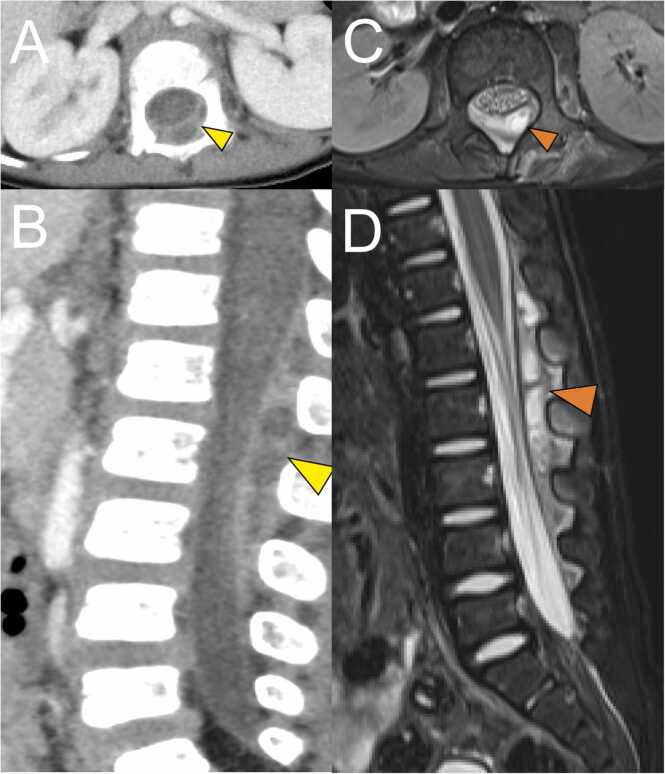
Contrast enhanced computed tomography (CT) imaging and magnetic resonance imaging (MRI) performed using a 3.0 T system. (A) Axial CT image. (B) Sagittal CT image. (C) Axial fat-suppressed T2-weighted MRI image. (D) Sagittal fat-suppressed T2-weighted MRI image. A multifocal cystic structure is visible dorsally on the dura at the Th12-L3 level in the spinal canal (yellow arrowhead in A, B; orange arrowhead in C, D). Contrast effects are present on the left lateral intervertebral foramen at the L1–2 level and on the erector spinae near the L1/2 intervertebral joint (A, B). A markedly high fat-suppressed T2-weighted image signal in the left intervertebral joint at the level of L1/2 is suggestive of fluid retention (C, D). The perivertebral foramen and erector spinae show high fat-suppressed T2-weighted image signals mainly at the L2 level, indicating inflammatory spillover (C, D). No abscess formation in the psoas major muscle or bone marrow edema in the vertebral body is visible.

On day 4 after hospitalization, the fever resolved and patient’s general condition improved. On the same day, blood tests showed a discrepancy in inflammatory markers. Improvement was observed in white blood cells (9520/µL) and CRP (5.66 mg/dL. However, there was prolonged activation of the coagulation-fibrinolytic system: fibrinogen, 904 mg/dL; FDP, 14.1 μg/mL; and D-dimer, 6.0 μg/mL. On day 5 after admission, blood and urine cultures showed negative results. Echocardiography and abdominal ultrasound did not reveal any abnormalities. Further examinations were performed on the same day. Contrast-enhanced thoracoabdominal computed tomography (CT) was performed, which revealed a large epidural mass in the spinal canal spanning the level of the 12th thoracic vertebra to the 3rd lumbar vertebra (Fig. 1A, C). Magnetic resonance imaging (MRI) was performed, which led to the diagnosis of facet joint arthritis with lumbar SEA (Fig. 1B, D). No significant compression of the spinal cord was observed. We discussed with the orthopedic surgeon whether surgical drainage of the abscess should be performed. Due to the consistent absence of neurological symptoms and resolution of fever, antibiotic therapy was continued without surgical treatment. Since blood cultures revealed negative results and the abscess was not drained, the causative agent of the SEA was unknown. The patient had achieved clinical improvement with Meropenem. Therefore, we continued him on Meropenem. No new symptoms developed. On day 17 after hospitalization, MRI was repeated. The SEA had disappeared almost completely. On day 21, the patient was switched from intravenous antimicrobial (meropenem) to an oral antimicrobial (cephalexin, 100 mg/kg/day). Regarding the choice of oral antibiotic, cephalexin was selected as a first-generation cephem antibiotic based on previous reports of treatment of SEA [4], [5]. The dosage was determined taking into account the severity of infection. The patient was subsequently discharged on day 24 after hospitalization. Oral antimicrobial therapy was continued for 2 weeks. Three months after onset, MRI revealed resolution of the epidural abscess and improvement in the high signal in the facet joint.

## Discussion

SEA is a rare condition and its classic symptoms, including back pain, fever, and neurological symptoms, are often incomplete [2], [5], [7], [8], [9], [10]. In this patient, there were no symptoms of local spinal tenderness such as weight-bearing refusal, pain on movement, or back pain. Fever was the only symptom. In the absence of such specific symptoms, it is difficult to suspect SEA. It is quite possible that the diagnosis may be delayed until the patient presents with neurological deficits such as weakness of the extremities, paralysis, or paraplegia that can be irreversible [4], [5], [7].

Regarding SEA, bacteria can reach the epidural space by direct invasion or hematogenous dissemination [4], [5]. Hematogenous spread is considered the most common cause of infection in children [4], [5]. Blood cultures often show positive results [3], [4], [5]. This patient had no history of lumbar or epidural injections. Therefore, hematogenous seeding was more likely than direct infiltration. However, this patient had negative blood culture results on admission. This result may have been influenced by the fact that the patient had already received oral antibiotic therapy prior to admission; even with SEA, blood cultures could be negative if antibiotics were administered prior to diagnosis [1], [4], [5]. Positive blood culture results may trigger suspicion of the presence of any abscess, including SEA. Even negative blood culture cannot rule out the presence of an abscess in the presence of prior antibiotic administration.

A total of 4–6 weeks of antibiotic therapy is required to treat SEA [2], [3], [5]. The missed diagnosis of SEA and ineffective antibiotic treatment could increase the likelihood of neurological sequelae [4], [5], [7]. Patients with SEA may quickly exhibit resolution of fever symptoms and improvement in blood parameters once antibiotic therapy is initiated, leaving the disease undiagnosed. If the physician is convinced that the patient's condition has improved and no further testing is performed, SEA would remain undiagnosed. As a result, only antibiotics are given for a period of time that is insufficient for resolving SEA. Thus, ineffective treatment without correctly diagnosing SEA may result in sequelae. To diagnose SEA, one must be aware of the possibility that an inflammatory focus may be present, even if the treatment response is favorable.

Contrast-enhanced CT or MRI is necessary for the diagnosis of SEA on the suspicion of the presence of an inflammatory focus [2], [4], [5]. In this case, there were no specific symptoms or risk factors and blood cultures results were negative. The patient quickly showed fever resolution. However, the patient exhibited markedly high CRP levels and persistent activation of the fibrinolytic coagulation system, including fibrinogen, FDP, and D-dimer. Since high CRP levels and persistent activation of the coagulation/fibrinolytic system were associated with severe inflammation and severe infection [11], [12], we suspected the presence of serious inflammatory foci. Contrast-enhanced CT has a risk of radiation exposure, and sedation is required for MRI studies. Nonetheless, we determined that the need to identify serious inflammatory foci in our patient outweighed these disadvantages; the SEA was subsequently diagnosed and adequate treatment was administered.

In summary, SEA should be strongly suspected in the differential diagnosis in children with fever of unknown origin, high levels of CRP, and persistent activation of the coagulation-fibrinolytic system. Although imaging studies have drawbacks, such as radiation exposure and the need for sedation, when encountered with a patient who presents with the abovementioned symptoms, appropriate imaging studies (contrast-enhanced CT and MRI) are necessary for early diagnosis and establishing a good response to treatment.

## Ethical approval

We obtained consent from the patient's guardian and approval from the hospital ethics committee for this case report.

## Consent

Written informed consent was obtained from the patient’s guardians for publication of this case report and accompanying images. A copy of the written consent is available for review by the Editor-in-Chief of this journal on request

## Funding

This research did not receive any specific grant from funding agencies in the public, commercial, or not-for-profit sectors.

## CRediT authorship contribution statement

**Yuta Nakamura**: Data curation, Writing – original draft, **Takahiro Namba**: Conceptualization, Data curation, Writing – original draft, **Momoko Sakurai**: Writing – review & editing, **Masato Yasui**: Writing – review & editing, Supervision.

## Declaration of Competing Interest

All authors have no conflicts of interest.
